# Unraveling the Initial Plant Hormone Signaling, Metabolic Mechanisms and Plant Defense Triggering the Endomycorrhizal Symbiosis Behavior

**DOI:** 10.3389/fpls.2018.01800

**Published:** 2018-12-17

**Authors:** Alberico Bedini, Louis Mercy, Carolin Schneider, Philipp Franken, Eva Lucic-Mercy

**Affiliations:** ^1^INOQ GmbH, Schnega, Germany; ^2^Department of Plant Physiology, Humboldt-Universität zu Berlin, Berlin, Germany; ^3^Leibniz-Institut für Gemüse- und Zierpflanzenbau Großbeeren/Erfurt, Großbeeren, Germany

**Keywords:** carbon partitioning, mycorrhizal fungi, phosphorus, physiology, phytohormones, plant defense, plant priming, signaling

## Abstract

Arbuscular mycorrhizal (AM) fungi establish probably one of the oldest mutualistic relationships with the roots of most plants on earth. The wide distribution of these fungi in almost all soil ecotypes and the broad range of host plant species demonstrate their strong plasticity to cope with various environmental conditions. AM fungi elaborate fine-tuned molecular interactions with plants that determine their spread within root cortical tissues. Interactions with endomycorrhizal fungi can bring various benefits to plants, such as improved nutritional status, higher photosynthesis, protection against biotic and abiotic stresses based on regulation of many physiological processes which participate in promoting plant performances. In turn, host plants provide a specific habitat as physical support and a favorable metabolic frame, allowing uptake and assimilation of compounds required for the life cycle completion of these obligate biotrophic fungi. The search for formal and direct evidences of fungal energetic needs raised strong motivated projects since decades, but the impossibility to produce AM fungi under axenic conditions remains a deep enigma and still feeds numerous debates. Here, we review and discuss the initial favorable and non-favorable metabolic plant context that may fate the mycorrhizal behavior, with a focus on hormone interplays and their links with mitochondrial respiration, carbon partitioning and plant defense system, structured according to the action of phosphorus as a main limiting factor for mycorrhizal symbiosis. Then, we provide with models and discuss their significances to propose metabolic targets that could allow to develop innovations for the production and application of AM fungal inocula.

## Introduction

Plant hormones, also called phytohormones, are organic compounds other than nutrients that are naturally produced by plant tissues in response to specific stimuli. They act spatially and temporally as endogenous signals able to organize all plant developmental stages (seed dormancy, seed germination, plant growth, flowering, etc.) by regulating at a very low dose various physiological functions. Plant hormones belong to the class of plant growth regulators, which group both natural and synthetic compounds that can regulate plant development (Sajjad et al., 2017). In addition to developmental regulation, they also play important roles in abiotic and biotic stress responses and in mutualistic interactions between plants and other organisms. Each of the plant hormones or plant growth regulators possesses specific functions, but they interact with each other antagonistically or cooperatively by complex crosstalks.

One of the most ancient and widespread mutualistic association concerns the endomycorrhizal symbiosis, in which particular soil fungi, called arbuscular mycorrhizal (AM) fungi, colonize the root of most (74%) plant families on earth (van der Heijden et al., 2015). These fungi belong to the Glomeromycotina (among the phylum Mucoromycota), regrouping at least 313 characterized species^1^. They were extensively studied for more than 60 years, as it was shown that they are key components of soil fertility. Many examples suggest to exploit AM fungi for promoting plant performances (growth, survival, and tolerance) as they can enhance nutrition (water and minerals), photosynthesis, protection against biotic and abiotic stresses, regulation of developmental processes (flowering, fruit and seed formation, rooting, etc.) and take part in soil structuration (Smith and Read, 2008). However, the wider use of mycorrhizal *inocula* in agricultural fields remains challenging, due to their cost, variability in term of quality and responses on plants as well as incompatibility with high available phosphorus (P) levels in soils (Vosátka et al., 2008; Ijdo et al., 2011; Berruti et al., 2016).

Arbuscular mycorrhizal fungi are obligate biotrophs, the completion of their life cycle requires the absolute presence of host plants that provide a specific habitat (as a physical support and a favorable metabolic frame) allowing fungal uptake and assimilation of likely several energy sources (sugars, probably lipids and maybe other unknown compounds) (Pfeffer et al., 1999; Helber et al., 2011; Rich et al., 2017). This definition remains vague because a formal demonstration of AM fungi development and production under axenic conditions is still lacking, feeding numerous debates within the mycorrhizologist community but illustrating a gap of knowledge in plant and fungal physiology. Consequently, the biology of AM fungi is probably among the most complex and difficult field of research in plant science and clues obtained are mostly indirect due to the presence of the host plant. Nevertheless, it can be confidently stated that P concentration as well as plant hormones, as signals targeting numerous biochemical reactions and gene regulation, can finally generate or not a favorable root tissue environment, driving the completion of the AM life cycle. Therefore, most approaches that create a range of conditions concerning P and phytohormones can represent valuable tools for understanding the regulation of AM fungi development *in planta* or *in vitro*.

Negatively correlated responses between P concentration in soil and mycorrhizal phenotype and function are well investigated (Smith and Read, 2008). However, the comprehensive action of P through plant metabolism beyond hormonal interplays is still not well understood. In recent years, there have been more studies published about the AM fungi responses to hormones. Evidence has shown that AM fungi are sensitive to plant hormones (*in planta* but also *in vitro*, in presence or not of plant, respectively, monoxenic and axenic conditions) and that they are able to produce at least some of them (see section “Phytohormones Influence the Mycorrhizal Symbiosis”). However, phytohormones represent only part of the signaling in AM symbioses and their concret translations on plant metabolic pathways, especially those involved in energetic partitioning (glycolysis, fermentation, REDOX potentials, lipid metabolism, TCA or mitochondrial respiration), remain poorly discussed. To fill in the knowledge gap of the definition of favorable or non-favorable plant metabolic framework for AM fungi is a major step toward understanding fungal needs, and can then provide insights about the mode of action of some elements such as phosphorus, as well as clues to artificially promote mycorrhizal performances (root colonization and AM responses on plants).

The use of mutant plants or hormonal plant pretreatments (discussed in sections below) suggests that the AM behavior within roots is consequently driven by the metabolic interplay initially set in the plant. The aim of this review is to provide a detailed theoretical picture, based on available knowledge, connecting plant hormones, plant metabolic pathways involved in cell energy, plant defense and AM development and growth. The key point is to define the physiological basis of the plant susceptibility to mycorrhiza prior to inoculation with AM fungi or AM root contact.

**FIGURE 1:**
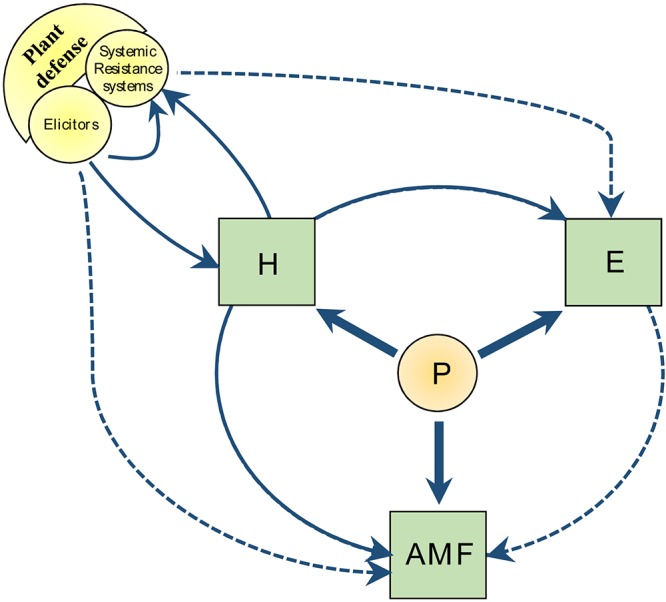
Overview of the metabolic crosstalk directions discussed in this review. Solid lines indicate state of the art according to the bibliographic survey. Dashed lines indicate perspectives based on interpretation of the metabolic crosstalk. P, phosphorus; H, plant hormone; E, energetic system (carbon partitioning, respiration, and REDOX potential); AMF, arbuscular mycorrhizal fungi.

The first section reviews the impact of 9 plant hormones, strigolactones (SL), abscisic acid (ABA), ethylene (ET), gibberellins (GA), salicylic acid (SA), jasmonate (JA), auxins (AUX), cytokinins (CK), and brassinosteroids (BR), on mycorrhizal behavior (root colonization, arbuscule formation and functionality). In the second section, we debate about the links between plant defense systems, compounds that induce a primed state (elicitors), plant hormone interplay, and AM fungal development. In the third section, we illustrate the impact of hormone interplay on plant energetic system [including photosynthesis, glycolysis, fermentation, lipid metabolism, tricarboxylic acid cycle (TCA), mitochondrial respiration, and REDOX potential]. As P represents a crucial criterion for the development and functionality of AM fungi, we describe here metabolic interplays under two hypothetic contrasting situations, low and high P, and we discuss whether the role of hormones and regulations within cells can be driven by P concentrations. Then, in a last section, we propose models that integrate signaling and plant energetic systems in mycorrhizal development, and strategies in which specific plant priming could be exploited as a tool to promote mycorrhizal performances (Figure 1).

## Phytohormones Influence the Mycorrhizal Symbiosis

**FIGURE 2:**
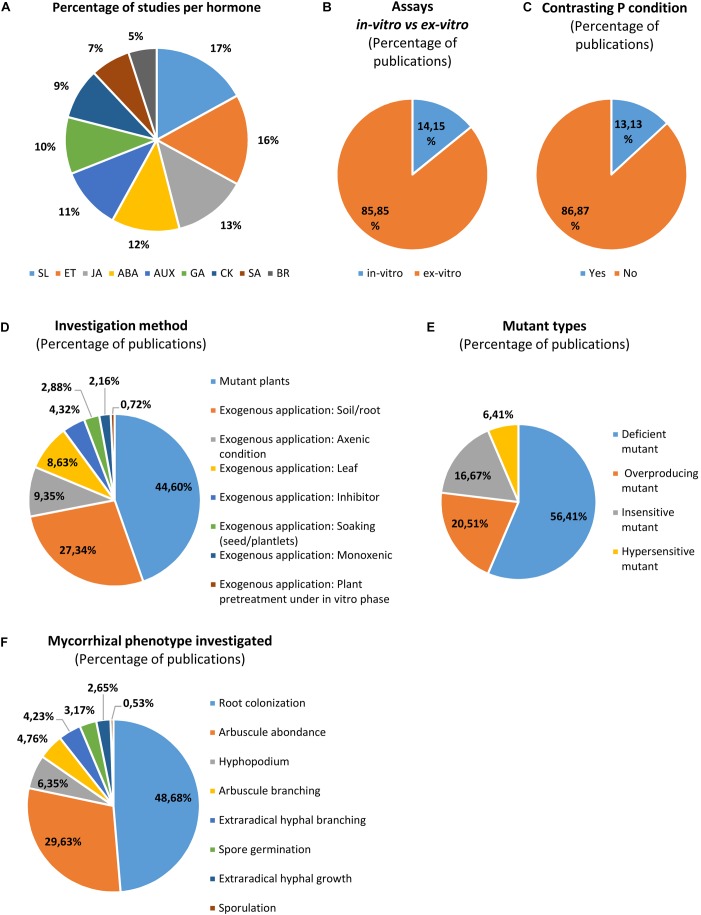
Overview of the methodologies used and the fungal phenotypic parameters analyzed to investigate hormone response in endomycorrhizal symbiosis. The percentage of publication studying each hormone **(A)** indicates that SL and ET are likely the most studied while SA and BR are the least investigated. Most of assays were conducted *in planta* (in greenhouse or growth chambers), **(B)** but few works were conducted under contrasting P levels **(C)**. The use of mutant plants is the most common method, but pharmacological approaches were also done with hormones (mostly by application on soil/root, in presence of mycorrhizal propagules) and inhibitors **(D)**. Less studies were conducted under monoxenic conditions or by hormonal plant pretreatment (which allow to limit direct interaction between hormones and AM inoculum) **(D)**. Hormone deficient mutants are most often used while hypersensitive mutant plants are less exploited **(E)**. Then, several AM phenotypic parameters were investigated, mostly root colonization and arbuscule abundance, but less data are available regarding other structures, in particular spore germination, extraradical hyphal growth and sporulation **(F)**. ABA, abscisic acid; JA, jasmonate; GA, gibberellins; SA, salicylic acid; SL, strigolactones; ET, ethylene; CK, cytokinins; IAA, auxins; BR, brassinosteroids. Note that more than one phytohormone was studied in some publications, creating redundancies which were counted to calculate the percentages presented in the figures (see Supplementary Material).

The knowledge of hormonal interplay in AM symbiosis is progressing for the past decade, but some phytohormones have been investigated more extensively than others. Up to now, SL and ET seem to be the most studied phytohormones, while SA and BR are the least investigated (Figure 2A). Overall most experiments use *ex vitro* (greenhouse/growth chambers) conditions (Figure 2B), and few trials were done in presence of various phosphorus concentrations (Figure 2C). Three main methodological approaches are classically used (Figures 2D,E): (i) exogenous application of hormones, (ii) use of mutant plants (deficient, overproducing, insensitive and hypersensitive), and (iii) application of hormone inhibitors. A list of a hundred references (non-exhaustive) surveyed for this review is available in a table (see Supplementary Material), in which we considered most AM fungal phenotypic parameters at different stages of the AM symbiosis development: (i) asymbiosis: propagule germination, but considering only AM spores and not mycorrhizal root fragments used as inoculum (studied in Gryndler et al., 1998), (ii) presymbiosis: hyphal growth and branching, and (iii) symbiotic stages: hyphopodium formation, root colonization, sporulation, arbuscule abundance and morphology (Figure 2F). The effects of phytohormones and their interplays on AM fungi and symbiosis were already described in several reviews (Foo et al., 2013a; Bucher et al., 2014; Fusconi, 2014; Gutjahr, 2014; Miransari et al., 2014; Pozo et al., 2015). Moreover, the regulation of the signaling between the symbionts and the molecular mechanisms beyond were detailed in a recent review (Liao et al., 2018). Therefore, we only briefly summarize actions of each hormone in the sub-sections below.

### Abscisic Acid (ABA)

Abscisic acid is a stress phytohormone belonging to the class of sesquiterpenes. It regulates negatively plant growth and controls stomatal closure, limiting water loss by transpiration (Świątek et al., 2004; Cutler et al., 2010). ABA also plays a role in interactions with phytopathogens by modulating tissue invasion depending on type of microorganism, site and time of infection (Ton et al., 2009).

Abscisic acid can play a role in all phases of AM symbiosis: exogenous ABA application promotes root colonization and arbuscule formation during the early symbiotic stage, but is also able to maintain spore dormancy during the asymbiotic phase (Herrera-Medina et al., 2007; Martín-Rodríguez et al., 2010, 2011, 2016; Mercy et al., 2017). Effects of soil applications of ABA seem dose dependent: in *Medicago truncatula*, low ABA concentration (5 × 10^-5^ M) promotes AM fungi development, arbuscule branching and abundance, while higher concentration (5 × 10^-4^ M) reduces the level of colonization (Charpentier et al., 2014). *In vitro* plant pre-treatment with ABA (applied in the culture medium) resulted in higher root colonization and arbuscule abundance (Mercy et al., 2017), which indicates that ABA creates a favorable metabolic context before contact with and colonization by AM fungi.

Majority of studies on ABA were conducted by using deficient or insensitive mutant plants. ABA-deficient *sitiens* tomato mutant harbored a reduced mycorrhizal susceptibility, with lower percentage of vesicules, arbuscules and fungal alkaline phosphatase activity compared to the corresponding wild type (Herrera-Medina et al., 2007; Aroca et al., 2008; García-Garrido et al., 2010; Martín-Rodríguez et al., 2010, 2011, 2016; Fracetto et al., 2017). The arbuscules were also not completely formed and fewer branches were counted (Herrera-Medina et al., 2007; Fracetto et al., 2017). Another ABA-deficient tomato mutant (*notabilis*) was also used, but except for one study in which mycorrhizal development and arbuscule intensity was reduced (Xu et al., 2018), no particular response on mycorrhizal colonization was noticed (Zsögön et al., 2008; Martín-Rodríguez et al., 2010; Fracetto et al., 2017) probably because ABA levels in the roots remained similar to the wild type (Martín-Rodríguez et al., 2010). AM fungi are able to produce ABA (Esch et al., 1994), and it was shown that AM fungi can increase endogenous ABA content in plant during colonization (Ludwig-Müller, 2010).

Abscisic acid signaling establishes complex crosstalks with other phytohormones, especially ET, SA, GA, and SL. Higher ET levels were found in roots of the tomato mutant *sitiens* (Herrera-Medina et al., 2007). Several studies described a negative effect of ET on AM fungal colonization (see below), but ABA can act independently of ET (Herrera-Medina et al., 2007; Martín-Rodríguez et al., 2010), confirming a direct role of ABA in AM fungi development. Furthermore, ABA levels in plants are interconnected with GA that negatively regulate later stages of AM fungi development in roots (see GA sub-chapter below; Foo et al., 2013a; Martín-Rodríguez et al., 2016). ABA downregulates gene expression involved in GA biosynthesis and increases GA catabolism (Martín-Rodríguez et al., 2016), but also represses biosynthesis and metabolic responses to ET and SA (Vlot et al., 2009). It is well accepted that the antagonistic signaling between ABA and GA targets specifically DELLA proteins, which belong to the GRAS family of plant regulatory proteins. DELLA proteins act as transcriptional suppressors in GAs signaling (Harberd, 2003) and are destabilized by GA (Silverstone et al., 2001) while ABA maintains DELLA integrity (Silverstone et al., 2001; Achard et al., 2006). DELLA was demonstrated as a central node connecting various signaling pathways activated during AM formation and positively regulating arbuscule formation (Floss et al., 2013; Foo et al., 2013a; Yu et al., 2014; Pimprikar et al., 2016). ABA and SL biosynthesis and/or signaling are probably connected because root contents of both hormones were positively correlated when comparing wild type with ABA-deficient mutant plants (*sitiens*, *flacca*, and to a lesser extent *notabilis*, López-Ráez et al., 2010).

In summary, it is consensus that ABA plays an enhancing role in the AM symbiosis, but we noticed that all the trials were conducted under low P concentrations (less than 50 ppm P) and no report evaluated the response of AM fungi to ABA under higher P level. Moreover, no study was conducted in order to connect natural endogenous ABA levels and mycorrhizal susceptibility from various plant species and/or cultivars. It is therefore not clear, if a dose dependence of ABA responses exists.

### Jasmonate (JA)

Jasmonate and its derivatives belong to a diverse class of lipid metabolites known as oxylipins (Wasternack, 2007; Mosblech et al., 2009) and are mainly involved in plant responses to biotic and abiotic stresses (Creelman and Mullet, 1997; Wasternack and Hause, 2002). JA is part of a signal transduction pathway activated by plant interaction with microorganisms (Pozo et al., 2004), leaf wounding (Schilmiller and Howe, 2005), and generally by abiotic stress conditions (Pedranzani et al., 2015). JA mediates higher transport rates of photosynthates to the roots (Babst et al., 2005; Schwachtje and Baldwin, 2008; Kaplan, 2012), and this might explain some of the positive effects on AM fungal colonization described below.

Exogenous application of JA has been shown to enhance AM fungal colonization (Regvar et al., 1996; Tejeda-Sartorius et al., 2008; Kiers et al., 2010; León-Morcillo et al., 2012) or to reduce it (Ludwig-Müller et al., 2002; Vierheilig, 2004; Herrera-Medina et al., 2008; Gutjahr et al., 2015). Repeated wounding or various abiotic stresses induce endogenous JA accumulation, and this was associated with higher AM fungal development (Landgraf et al., 2012; Pedranzani et al., 2015). Tests with various JA concentrations under contrasting P showed a strong dose dependence of the AM fungal response: mycorrhizal colonization is enhanced preferentially at 0.5 mM JA under high P (75 kg P Ha^-1^ year^-1^) but decreased at 5 mM JA under low P (25 kg P Ha^-1^ year^-1^; Kiers et al., 2010). However, no fungal phenotype response from P levels was obtained in JA-deficient rice line *cpm2* (Gutjahr et al., 2015). Presence of AM fungi was associated with up-regulation of oxylipins biosynthesis, and JA-deficient tomato lines *spr-2* and *def-1* showed reduced AM fungal colonization (Tejeda-Sartorius et al., 2008; León-Morcillo et al., 2012; Song et al., 2013, 2014, 2015). In particular, the genes encoding 9-lipoxygenases (9-LOXs) involved in JA biosynthesis seem to have a role in regulation of AM fungal development and in restriction of pathogen spreading (Blée, 2002; Vellosillo et al., 2007; Mosblech et al., 2009). A reduction of root colonization and arbuscule abundance of *Rhizoglomus irregulare* in *M. truncatula* was observed when a gene that encodes for allene oxidase cyclase (AOC), which is involved in JA biosynthesis, was suppressed by a root application of a *MtAOC1antisense* construct (Isayenkov et al., 2005). Repeating this experiment in *M. truncatula* with an *AOC1* RNAi and an overexpression construct did not show any impact on AM fungal colonization or on mycorrhiza-induced resistance (Hilou et al., 2014). Moreover, a JA-signaling perception mutant *jai-1* (jasmonic acid insensitive 1) was shown to be associated with higher AM fungal colonization and arbuscule formation (Herrera-Medina et al., 2008), but this response was not reproductible (Song et al., 2013).

Increased JA levels in roots of mycorrhizal plants were observed several times in different species (Vierheilig and Piché, 2002; Meixner et al., 2005; Stumpe et al., 2005; Hause et al., 2007). JA is also known to be linked with other phytohormones. It especially induces ABA biosynthesis, but ABA as well as SL are also required for JA production (Adie et al., 2007; Torres-Vera et al., 2014; de Ollas et al., 2015; de Ollas and Dodd, 2016; Haq et al., 2017). It is, moreover, well known that JA and SA are antagonists (see sections “Relationship Between Plant Defense Components and Mycorrhizal Symbiosis” and “Hormonal and Energetic Regulation of Plant Metabolism Under Contrasting P Conditions” below).

To conclude, JA effects are less clear but observations favor the hypothesis that JA is a promoting regulator of the AM symbiosis. Nevertheless, the contrasting data suggest that JA response is strongly sensitive to its endogenous concentrations, which can also modulate responses to exogenous JA application. Moreover, environmental factors (such as P) can interfere with JA perception, making interpretations quite delicate. This is well illustrated by the lack of mycorrhizal responses when JA is applied at low P, probably because endogenous JA levels are already high (Khan et al., 2016), but plant performance improvement can be observed at high P (Kiers et al., 2010). The effect of JA on AM fungi has not been studied *in vitro*, and if AM fungi are able to produce this phytohormone remains to be investigated.

### Auxin (AUX)

Auxins are mainly formed from tryptophan and indole-3-acetic acid (IAA) is the most abundant auxin. AUX regulate various aspects of plant growth and development, such as phototropism, geotropism and cell elongation and polarity (Benková et al., 2003). IAA regulates the development of lateral and secondary roots, which represent the preferential sites for AM fungal colonization (Kaldorf and Ludwig-Müller, 2000; Yao et al., 2005).

Observation *in planta* indicated that applications of various auxins (IAA, indole-3-butyric acid, 2,4-dichlorophenoxyacetic acid and 1-naphthaleneacetic acid) are able to promote AM fungal spread and arbuscule abundance (Dutra et al., 1996; Niranjan et al., 2007; Etemadi et al., 2014; Liu et al., 2016). In axenic conditions, it was also shown that IAA decreased both spore germination and subsequent hyphal germ tube growth (Fernández Suárez et al., 2015). The use of P-chlorophenoxyisobutyric acid, an IAA inhibitor, negatively affects the number of fungal entry points and further intraradical AM fungal development (Liu et al., 2014). AUX effects were also studied under monoxenic conditions. Mohan Raj et al. (2016) tested various indole-3-butyric acid and IAA concentrations (alone and combined) with *Daucus carota* transformed roots (*in vitro*) inoculated with *R. irregulare*, and shown a slight decrease in both root colonization and spore numbers.

Studies with the auxin (IAA)-deficient *bushy* mutant (Symons et al., 1999, 2002) is associated with a reduction in the AM fungal colonization but without further alteration of AM fungal structures inside the roots (Foo, 2013). Moreover, tomato auxin-resistant *diageotropica* (*dgt*) mutant shows lower AM fungal development in both monoxenic and *ex vitro* conditions (Hanlon and Coenen, 2011), although this was not always reproducible (Zsögön et al., 2008). The AM fungal response to auxin appears even more complex, as the auxin hyper-transporting *polycotyledon* (*pct*) mutant fails to generate an AM symbiosis monoxenically with root organ culture, but shows increased colonization in *ex vitro* plants, and authors suggested the existence of shoot-derived factors that modulate auxin action (Hanlon and Coenen, 2011). In another approach, microRNAs were used, which are non-coding RNAs that target particular genes and impair their expression. In this way, suppression of auxin-related signaling was achieved by overexpression of microRNA393, known to post-transcriptionally modulate the expression of the auxin receptors TIR1 and several AFBs (Navarro et al., 2006, 2008; Parry et al., 2009; Vidal et al., 2010). This strongly inhibited AM fungal colonization and arbuscule abundance and morphology in several plant species (Etemadi et al., 2014).

Regarding the interaction with other hormones, it has been proposed that auxins regulate positively SL biosynthesis genes (Foo et al., 2005; Johnson et al., 2006; Agusti et al., 2011), thereby participating in mycorrhizal development (Foo et al., 2013b). Moreover, auxin/cytokinins antagonism in root is very well known (Moubayidin et al., 2009), and a recent publication demonstrated that CK produced in roots are detrimental for AM fungi development (Cosme et al., 2016).

Several authors described increased IAA levels in mycorrhizal roots (Meixner et al., 2005; Ludwig-Müller and Güther, 2007; Fiorilli et al., 2009; Hanlon and Coenen, 2011; Liu et al., 2018) suggesting an involvement of IAA signaling in the first stages of colonization. Moreover, auxin-like molecules or IAA were found in small quantities in AM fungal spores (Barea and Azcón-Aguilar, 1982; Barea, 1986; Ludwig-Müller et al., 1997), but AUX were not detected in hyphae (Jentschel et al., 2007). Recent survey of the *R. irregularis* genome indicates that typical auxin biosynthesis genes are lacking (Tisserant et al., 2013), which may interrogate the (plant?) origin of AUX found in mycorrhizal structures, shown in previous publications.

To conclude, a positive effect of AUX seems to dominate, and is therefore classified as an AM-promoting hormone.

### Strigolactone (SL)

Strigolactones are terpenoid lactones derived from carotenoids which were originally discovered in root exudates, as they stimulate seed germination of parasitic plants like *Striga* (Mangnus and Zwanenburg, 1992). It turned later out that they also induce hyphal branching of AM fungal hyphae by affecting different molecular and cellular processes (Akiyama et al., 2005; Besserer et al., 2006). Despite SL being a recently discovered phytohormone, it is probably the most studied one in endomycorrhiza (Figure 2).

*In planta*, exogenous application of GR24 (a synthetic analog of SL) promotes mycorrhizal development in wild-type but also in SL-deficient plant mutants (Gomez-Roldan et al., 2007; Breuillin et al., 2010; Balzergue et al., 2011; Illana et al., 2011; Yoshida et al., 2012; Foo et al., 2013b). However, this induction appears to occur only under low P concentrations (Breuillin et al., 2010; Balzergue et al., 2011). SL application is unable to trigger mycorrhizal development in non-host plants such as *Arabidopsis thaliana*, *Spinacia oleracea*, *Lupinus polyphyllus*, or *Fagopyrum esculentum* (Illana et al., 2011). *In vitro* studies showed that SLs increase fungal metabolism, as application of synthetic analogs (GR24 and GR7) under axenic conditions was shown to activate mitochondrial differentiation, number and activity toward fungal cytochrome oxidase (COX) pathway with an increase in NADH and ATP during the pre-symbiotic phase in hyphea, and goes concomitantly with higher hyphal branching (Akiyama et al., 2005, 2010; Besserer et al., 2006, 2008; Balzergue et al., 2011; Kretzschmar et al., 2012). Moreover, spores in contact with SLs harbor higher germination rates in a shorter time (Besserer et al., 2006, 2008). In another work, Genre et al. (2013) demonstrated that GR24 is also able to stimulate production and release of Myc factors from spore exudates, which in turn induce Ca^2+^-spiking in the plant.

Strigolactone-deficient mutants or transgenic lines harboring constructs for RNAi-mediated silencing of genes participating in SL biosynthesis or signaling showed lower mycorrhizal colonization levels than the corresponding controls in tomato, rice, pea and petunia (Gomez-Roldan et al., 2008; Breuillin et al., 2010; Koltai et al., 2010; López-Ráez et al., 2010; Vogel et al., 2010; Illana et al., 2011; Kohlen et al., 2012; Kretzschmar et al., 2012; Yoshida et al., 2012; Foo et al., 2013b; Guillotin et al., 2017; Kobae et al., 2018). SL-insensitive mutant plants possess reduced to neutral responses on mycorrhizal rate, depending of the mutant type and plant variety (Yoshida et al., 2012).

Strigolactone interplays displayed positive crosstalk with ABA and AUX (see section above), and both of them have been recognized to stimulate mycorrhizal growth. The consensus for the role of SLs in mycorrhiza is clear, and can be confidently classified as promoter. However, the production of SL by AM fungi is not known.

### Brassinosteroid (BR)

Brassinosteroids represent a class of phytohormones defined as growth promoters (Kosová et al., 2012). They modulate plant development through important processes like cell elongation, cell division and cell differentiation. Furthermore, they are involved in defense against pathogens and abiotic stresses (Zhu et al., 2013).

Foliar application of epibrassinolid was shown to enhance slightly mycorrhizal colonization (Tofighi et al., 2017). Inoculation of the brassinosteroid-deficient pea mutant *lk* (Reid and Potts, 1986; Ross et al., 1989), which shows a strong reduction in BR content (Nomura et al., 2004), resulted in a strongly reduced total root colonization and a lower amount of arbuscules compared to the wild type (Foo et al., 2016). Tomato mutants defective in BR biosynthesis were analyzed with respect to mycorrhizal symbiosis and showed decreased mycorrhization (Bitterlich et al., 2014a,b). However, the leaky brassinosteroid-deficient *lkb* mutant pea did not induce a similar depressed fungal spread within roots (Foo et al., 2013a) suggesting that the reduction of BR levels must be severe to impact the AM symbiosis.

It is known that BRs can antagonize the plant innate immune response (Bajguz and Hayat, 2009; De Vleesschauwer et al., 2012; Nahar et al., 2013), and act together with other phytohormones in the case of interactions with biotrophs/necrotrophs (Saini et al., 2015). The later seems to be true also in the case of mycorrhizal interactions. The model proposed by Foo et al. (2016) suggested indeed that BR interacts negatively with ET in regulation of mycorrhizal behavior. However, how BR can interact with other hormones within mycorrhizal symbiosis remains to be elucidated. BRs also interact with the carbohydrate metabolism, and it was supposed that improvement of mycorrhizal development by BRs is based on a physical interaction between proteins involved in BR biosynthesis and signaling and a sucrose transporter (Bitterlich et al., 2014a,b).

In conclusion, BR is still poorly studied with respect to the AM symbiosis, but first data suggest that it acts as a promoter of mycorrhizal colonization. Nevertheless, more investigations are needed like, e.g., the impact of exogenous application of BR on the mycorrhizal symbiosis under *in vitro* and *ex vitro* conditions and the ability of AM fungi to synthetize BRs.

### Ethylene (ET)

Ethylene is a gaseous plant hormone; it plays an important role in plant signaling with fundamental effects on plant growth and development (fruit ripening, stem and root elongation inhibition, flowering, seed germination and leave senescence) as well as defense (van Loon et al., 2006a; Lei et al., 2011).

Exogenous soil and foliar treatments with ET or its commercial analogous ethephon have been shown to impair mycorrhizal colonization (Azcón-Aguilar et al., 1981; Morandi, 1989; Geil et al., 2001; Geil and Guinel, 2002; Foo et al., 2016) and to reduce arbuscule abundance (Geil et al., 2001; Geil and Guinel, 2002; Foo et al., 2016) with effects on arbuscule branching (Geil et al., 2001; Geil and Guinel, 2002). Interestingly, ET applied in specific amounts is able to promote mycorrhizal development under high P (Torres de Los Santos et al., 2016). Dose dependency was also shown under axenic conditions, where ET promoted hyphal growth and spore germination at low dosage, but inhibited it at a dosage higher than 0.2 ppm (Ishii et al., 1996).

Several studies using mutants have been conducted to determine the ET effect on AM symbiosis, but results were contradictive. In ET-overproducing tomato plants (*epinastic*), mycorrhizal colonization and arbuscule abundance were impaired at low P (Zsögön et al., 2008; Torres de Los Santos et al., 2011, 2016; Fracetto et al., 2013, 2017), but increased at higher P (Torres de Los Santos et al., 2016). In ET-insensitive mutant plants (tomato *never ripe*, tobacco *etr1* and pea *ein2* mutants), mycorrhizal colonization was found to be repressed (Zsögön et al., 2008), improved (Penmetsa et al., 2008; Martín-Rodríguez et al., 2011) or remained constant (Riedel et al., 2008; Fracetto et al., 2013; Foo et al., 2016; Torres de Los Santos et al., 2016). Similar inconsistent observations were found with ET-deficient tomato plants, with inhibited (Martín-Rodríguez et al., 2011), enhanced (Torres de Los Santos et al., 2011, 2016) or without effect on mycorrhizal root growth compared to wild-type plants (Riedel et al., 2008). No changes were recorded in the mycorrhizal development within an ET-hypersensitive tomato line (Martín-Rodríguez et al., 2011). Conflicting observations were also found for effects on biomass. In ET-deficient tomato, plant root growth was reduced (Martín-Rodríguez et al., 2011), enhanced (Torres de Los Santos et al., 2011, 2016) or remained unaffected by mycorrhization. Mycorrhizal development remained unaffected in ET-deficient (silencing of *coi1*) and ET-insensitive (*etr1*) tobacco plants, but mycorrhizal growth responses were strongly enhanced in both mutants (Riedel et al., 2008).

The relationship between endogenous ET level in roots and the mycorrhizal behavior is also not always clear: (i) negative correlation was observed within pea E107 (brz) (Resendes et al., 2001; Morales Vela et al., 2007) or in ABA-deficient *notabilis* and *sitiens* tomato mutant plants (Herrera-Medina et al., 2007; Martín-Rodríguez et al., 2010); and (ii) even more confusing, a positive correlation between endogenous ET root content and mycorrhization was noticed in both, ET-overproducing (*epinastic*) and ET-deficient (*rin*) tomato mutants, while harboring curiously, respectively, lower and higher ET content compared to the wild type (Torres de Los Santos et al., 2016).

Interactions between ET and other phytohormones was demonstrated. ABA-deficient *notabilis* and *sitiens* mutant plants harbor elevated ET levels in roots and an impaired mycorrhizal development, while exogenous ABA application reduced ET concentrations (Sharp et al., 2000; Herrera-Medina et al., 2007; Martín-Rodríguez et al., 2011; Fracetto et al., 2017). This suggests an antagonistic interaction between ABA and ET. ET was also shown to be negatively regulated by BR (Morales Vela et al., 2007), and ET-insensitive *ein2* mutant harbored reduced GA but elevated IAA levels (Foo et al., 2016). This corresponds with another study showing that application of 1-aminocyclopropane carboxylic acid (ACC), a precursor of ET, decreases free IAA content in roots (Negi et al., 2010).

Ethylene is likely the most problematic hormone to study as illustrated by the conflicting reports. Discrepancies with the use of mutant plants are likely due to the plant species or to the experimental conditions, which consequently limit formal interpretation. It was also suggested that ET has to reach a threshold before it influences AM fungal colonization and might explain why the mutants did not always show the same outcomes (Foo et al., 2016). The difficulties to interprete ET responses may also be due to the gaseous nature of ET. Its synthesis is stoichiometrically correlated with HCN by-production in plants (Peiser et al., 1984; Grossmann, 2003) and exogenous ET application stimulates endogenous ET biosynthesis. Versatile responses may be therefore attributed to the action of both, ET and HCN and the fine-tuning of their concentrations. Even though HCN is usually detoxified rapidly by plants (Miller and Conn, 1980), high local concentrations (up to 350 μM) can occur (Mizutani et al., 1988), with known impact on mitochondrial respiration (Siegień and Bogatek, 2006). Considering the aerobic nature of AM fungi, it is possible that high HCN concentrations are detrimental for fungal spread in roots but may promote locally arbuscule formation and functionality as it was observed with KCN application (Mercy et al., 2017). Moreover, HCN can elicit responses similar to ET when applied at low concentrations (McMahon Smith and Arteca, 2000). ET production by AM fungi remains unknown. Therefore, to understand the role of ET in the mycorrhizal symbiosis appears very challenging. In summary, we here state (with a risk) that ET negatively affects the mycorrhizal symbiosis.

### Cytokinin (CK)

Cytokinins are a class of diverse phytohormones formed by N^6^-substituted purine derivatives. CKs regulate several aspects of plant development such as shoot cell division and development and mineral uptake of roots (Werner and Schmülling, 2009; Kieber and Schaller, 2014).

Few studies investigated the response of AM to CK application: kinetin/kinetin riboside did not impact mycorrhizal development (Xie et al., 1998; Rabie, 2005) while 6-benzylaminopurine decreased it (Bompadre et al., 2015). Application of kinetin riboside on *Glomus clarum* spores (axenic conditions) promoted spore germination and germ tube growth (Fernández Suárez et al., 2015). AM fungi seems able to produce CK or CK-like hormones (Barea and Azcón-Aguilar, 1982). Many publications reported changes of endogenous CK content (ranging most often from increase to sometimes decrease) following AM fungi inoculation (Cosme et al., 2016).

Experiments with the CK-insensitive *bushy root* (Zsögön et al., 2008) or the CK receptor mutant *cre1* (Laffont et al., 2015) showed no impact on AM fungal colonization patterns. Laffont et al. (2015), however, stated that this is consistent with the limited transcriptional response of CK-regulated genes in roots. A tobacco transgenic line with low CK production showed increased AM colonization (Cosme and Wurst, 2013), but this effect was not reproducible. Indeed, when those plants were inoculated with two other AM fungal strains, the lower CK content was associated with impaired mycorrhizal colonization (Cosme et al., 2016). This was supported by another study, where increased colonization has been observed in a CK-overproducing pea mutant *E151* (Jones et al., 2015).

There exist antagonistic interplays between CK and ABA, as exogenous application of ABA can reduce CK content and perception (Werner et al., 2006; Tran et al., 2007; Großkinsky et al., 2014) and vise-versa (El-Showk et al., 2013; Guan et al., 2014). CKs also act synergetically with SA and GA, with consequences for the systemic acquired resistance (SAR, Großkinsky et al., 2014). CK perception and content is also regulated by P, and was shown to be repressed under P starvation (Salama and Wareing, 1979; Horgan and Wareing, 1980; Franco-Zorrilla et al., 2002, 2005; Rouached et al., 2010). Moreover, CKs act antagonistically to auxins in control of lateral root development (Moubayidin et al., 2009). Taking these findings into account, cytokinin biosynthesis seems not to be part of favorable condition frame (P level) and physical support (lateral roots) for possessing a positive influence on mycorrhizal development.

Formal interpretation of CK impact on mycorrhizal symbioses from existing literature remains delicate due to the low number of trials, different plant species and P concentration used in these tests, and the ability of AM fungi to produce this hormone (Supplementary Material). To conclude, the consensus of this phytohormone is not obvious but data suggest that CK does not play a major role in endomycorrhizal symbiosis but may act negatively mostly indirectly, *via* its impact on root system, its crosstalks with other hormones and its interplay with carbohydrate metabolism.

### Gibberellin (GA)

Gibberellins are a class of phytohormones synthesized from geranylgeranyl diphosphate. They move relatively free from shoots to roots promoting plant growth including stem elongation, flowering and inhibit leaf and fruit senescence (Swain et al., 2005; Wang and Irving, 2011; Claeys et al., 2014).

Many studies show that soil or leaf applications of GA reduce colonization and/or arbuscule abundance in several plant species (El Ghachtouli et al., 1996; Floss et al., 2013; Foo et al., 2013a; Yu et al., 2014; Martín-Rodríguez et al., 2015, 2016; Takeda et al., 2015; Khalloufi et al., 2017). In accordance, application of the GA biosynthesis inhibitor prohexadione calcium promotes mycorrhizal development (Martín-Rodríguez et al., 2015, 2016). GA seems able to promote spore germination under axenic conditions (Mercy et al., 2017) and AM fungi can produce this phytohormone (Barea and Azcón-Aguilar, 1982; Strzelczyk and Pokojska-Burdziej, 1984).

Several studies with overexpressing or deficient mutant plants for GA-biosynthesis and GA-signaling indicated a negative role of GA for arbuscule formation and development, emphasizing a negative impact on late stage of development (Floss et al., 2013; Foo et al., 2013a; Martín-Rodríguez et al., 2015, 2016). Growth regulator interconnection converges toward the stabilization status of DELLA proteins, which are integrated in abiotic and biotic stress (Zentella et al., 2007; Davière and Achard, 2013; Yu et al., 2014). GA was demonstrated as reciprocal antagonist with ABA and JA (Brenner et al., 2005; Greenboim-Wainberg et al., 2005; Razem et al., 2006; Yang et al., 2012; Heinrich et al., 2013; Shu et al., 2018) in almost all plant physiology aspects (plant defense reaction, seed dormancy and germination, growth, etc.). It appears that DELLA proteins negatively control all GA responses, and the degree of its stability in cell depends on the GA/ABA-JA ratio. Thus, ABA (Achard et al., 2006) and JA (Yang et al., 2012; Wild and Achard, 2013) stabilize or promote the DELLA complex, positively associated with arbuscule formation, while GA induces its ubiquitin-proteasome degradation associated with collapsed arbuscules (Floss et al., 2013; Bucher et al., 2014; Yu et al., 2014; Martín-Rodríguez et al., 2015). Moreover, GA-induced degradation of DELLA proteins enhances SA signaling, increasing plant resistance to biotrophic microorganisms (Navarro et al., 2008; Wasternack and Hause, 2013) like AM fungi. The consensus for GA is therefore well defined as a negative regulator of the mycorrhizal symbiosis.

### Salicylic Acid (SA)

Salicylic acid is a phenolic compound classified as plant hormone a decade ago (Eraslan et al., 2008; Shi et al., 2009). It regulates many aspects of plant physiology, such as growth, ion uptake and chlorophyll content (Singh and Usha, 2003; Eraslan et al., 2008; Belkhadi et al., 2010). Furthermore, SA has long been known to play a major role in reducing plant stress, increasing the antioxidant activity (Shi et al., 2009) and promoting activation and modulation of plant defense responses, especially in interaction with biotrophic pathogens (Beckers and Spoel, 2006; Lu, 2009).

Exogenous applications of SA was shown to reduce mycorrhizal development, at least during the first weeks (Blilou et al., 2000; Costa et al., 2000; Özgönen et al., 2001; de Román et al., 2011), but neutral responses were also observed (Ludwig-Müller et al., 2002; Ansari et al., 2016). AM colonization can also increase following soaking seeds with SA (Garg and Bharti, 2018). Moreover, tobacco SA-overproducing mutant *CSA* and SA-deficient *nahG* showed, respectively, reduced and enhanced root colonization in the first days following fungal penetration (Herrera-Medina et al., 2003). Similarly, the Myc^-^ pea mutant P2 was found to accumulate higher SA concentration in roots (Blilou et al., 1999).

Salicylic acid seems to affect mycorrhizal development mainly at early stages. This effect seems transitory, probably due to the ability of the fungus to modulate the plant defense response further (Dumas-Gaudot et al., 2000; Campos-Soriano et al., 2010; de Román et al., 2011). Regulation of SA on other hormones within mycorrhizal symbiosis remains to be elucidated, but some connections can be found in relation with plant defense system (discussed in sections below). However, it can be stated as consensus that SA act as inhibitor of the mycorrhizal symbiosis. It is not yet known if AM fungi can synthetize this hormone, and effect of SA on mycorrhizal behavior under axenic conditions remains to be investigated.

## Relationship Between Plant Defense Components and Mycorrhizal Symbiosis

Hormone signaling is tightly linked with defense pathway activation *in planta* (Bonneau et al., 2013). Contact with pathogens, beneficial microorganisms, natural and synthetic compounds or presence of abiotic stress trigger at various physiological, transcriptional, metabolic and epigenetic levels an unique plant state called “priming,” resulting in establishment of induced defense mechanisms (Conrath et al., 2006; Mauch-Mani et al., 2017). Usually, but non-exclusively, two main antagonistic induced responses are engaged in plants, depending on the priming signal (named elicitor): systemic acquired resistance (SAR) and induced systemic resistance (ISR). The SAR response is induced by biotrophic pathogens (Ton et al., 2009; Thakur and Sohal, 2013) and involves SA accumulation, which mediates the activation of pathogenesis-related (PR) genes (Durrant and Dong, 2004). PR proteins are known especially for their antifungal activity based mainly on the hydrolytic capacity toward fungal cell wall components (Edreva, 2005). The ISR response, instead, is induced by necrotrophs or plant growth-promoting rhizobacteria (PGPRs) and involves JA and ET signaling without modification of defense gene expression (Pieterse et al., 1996, 2002). Specifically, ISR is based more on enhanced sensitivity to these plant hormones rather than to an increase in their production (Pieterse et al., 1998; Pieterse and van Loon, 2004; De Vleesschauwer et al., 2006). The role of ET remains somewhat difficult to define as a strict ISR component: it was shown originally to be required in ISR (Pieterse et al., 1998), but it contributes also to SAR by the induction of PR genes during the hypersensitive response against tobacco mosaic virus as one of the mobile signals, that SA is not in this case (Kuć, 2006; van Loon et al., 2006b). The mode of action of ET largely depends on the moment when it is produced, and ET treatment of plants can lead to opposite effects (i.e., before or after pathogen infections, van Loon et al., 2006b). Finally, many studies showed that almost all the plant hormones could participate to different extent in induced plant resistance (Pieterse et al., 2012). For example, additionally to abiotic stresses, ABA has a role in plant pathogen interactions (Fan et al., 2009; Cao et al., 2011). Emerging evidences state importance of ABA in plant defense system, with suppression of SAR induction and involvement in SA-SAR-mediated signaling (Yasuda et al., 2008; Jiang et al., 2010; Kusajima et al., 2010) but its potential role in ISR establishment is less clear as it can also counteract JA/ET defense related pathways (Cao et al., 2011).

Although the knowledge on plant pathogen interactions made important progress in the last years, classification of many important hormones involved as part of either ISR or SAR system remains incomplete (Pieterse et al., 2012). Moreover, interactions between plants and beneficial microorganisms partially exploit the same defense related pathways. Firstly, as shown by Güimil et al., 2005, there is a 40% overlap between genes responding to AM fungi and pathogen agents in rice. Although these responses are temporally and spatially limited in mycorrhizal symbiosis compared to phytopathosystems, this suggests that the plant defense system may play a role in the establishment and control of the endomycorrhizal symbiosis (Dumas-Gaudot et al., 1996; García-Garrido and Ocampo, 2002). Secondly, several authors suggested that AM fungi implement ISR in plant, during the first colonization stages (Pozo et al., 2002; Hause and Fester, 2005; Hause et al., 2007; Pozo and Azcón-Aguilar, 2007; Kapoor et al., 2008; Pieterse et al., 2014) but also that PGPRs, known to elicit ISR, can increase the mycorrhizal development (Alizadeh et al., 2013). By contrast, SAR system seems to generate a non-favorable metabolic context for AM fungi, since the use of SAR elicitors can lead to inhibition of mycorrhizal development (Faessel et al., 2010; de Román et al., 2011; Bedini et al., 2017) sharing therefore similarities with biotrophic pathogens (Delaney et al., 1994). As a point, while glycerol-3-phosphate is converted into glycerol and phosphate under P-deficient conditions (Hammond et al., 2004), it probably accumulates under P-sufficient plants increasing SAR stimulation potentials via SA (Chanda et al., 2011; Shah and Zeier, 2013).

## Hormonal and Energetic Regulation of Plant Metabolism Under Contrasting P Conditions

Phytohormones act as messengers within the plant, which syntheses are usually regulated by various stimuli. However, their actions on mycorrhizal behavior should be connected to a specific metabolic plant state, favoring or not mycorrhizal development beyond their energetic needs. In this section, we discuss the connection between the metabolic context and hormone interplay under two contrasting situations of P level, supporting (low P) and inhibiting (high P) mycorrhizal colonization.

Plants acquire P by two different pathways. The first one, common for all plants, is called the direct pathway by which P is collected directly *via* the surface plant roots. The second one, called mycorrhizal pathway, is builded by the presence of mycorrhizal fungi which are able to uptake and transfer the P from soil to the root *via* the mycelium. In both cases, P uptake and transfer involves an active translocation mediated by H^+^-ATPases which create a proton motive force allowing P entering the cell *via* Pi/H^+^ symporters localized in the rhizodermis or the root hairs (direct pathway) or in the periarbuscular membrane at the arbuscule branch domain (mycorrhizal pathway, Smith and Smith, 2011b; Młodzińska and Zboińska, 2016).

### Mycorrhizal Fungal Growth Has a Preference for a Metabolic Context Related to P Stressed Plants

#### Plant Respiration Under Low P

In this review, low available P is defined as a concentration belonging or being close to plant P starvation, which favors mycorrhizal development within roots (Smith and Read, 2008). Frequently, natural soils have P concentrations below 1 μM. Furthermore, P absorption by the roots results in a rapid depletion zone due to the low mobility of this ion (Marschner, 1995). This consequently engenders plant P starvation. Plant primary metabolism is then altered, as P stress induces a shift in plant respiration with reduced plant capacity to produce ATP (Theodorou and Plaxton, 1993; Plaxton and Tran, 2011) and is associated with deficient photosynthesis (Fredeen et al., 1990; Ghannoum and Conroy, 2007). Thus, plants undertake a series of metabolic adaptations in order to conserve the use of P, such as reduction of cell energetic potentials associated with plant growth depression, increased efficiency in P utilization, and remobilization of internal P and mitochondrial bypass P-requiring steps (Schachtman et al., 1998; Plaxton and Carswell, 1999; Raghothama, 1999; Uhde-Stone et al., 2003a,b; Plaxton and Tran, 2011).

One of the first adjustments at P deficiency is the re-organization of the electron chain transport within plant mitochondria. In the last steps of respiration, electrons provided by the TCA cycle are typically transported along the mitochondrial complexes I, II, III, and complex IV, the cytochrome oxidase pathway (COX). Complexes I, III, and IV constitute proton pumps during the electron transport, leading to the formation of a proton gradient between the mitochondrial matrix and the intermembrane space (Alberts et al., 2008). The gradient generated by the complexes is then used by ATP-synthases to produce ATP (Nakamoto et al., 2008). In cases of stress like P starvation, electrons are redirected to another terminal oxidase that is part of the alternative oxidase pathway (AOX). This pathway, present in plants and fungi, is sited between complexes II and III and catalyzes the reduction of oxygen into water, resulting in a lower intermembrane proton gradient and reduced ATP yield (Sluse and Jarmuszkiewicz, 1998). AOX pathway has been described as a pivotal element able to maintain the cell metabolic homeostasis, participating to the carbon metabolism flexibility (Gomez-Casanovas et al., 2007; Gandin et al., 2009; Leakey et al., 2009; Vanlerberghe, 2013). For this reason, it has been proposed as an important marker for plant acclimatization to stress conditions (Arnholdt-Schmitt et al., 2006; Clifton et al., 2006). Finally, AOX pathway was also proposed to play a role in AM spore dormancy and germination, as well as, AM fungal behavior *in planta*, influencing both colonization and arbuscules functionality (Besserer et al., 2009; Campos et al., 2015; Mercy et al., 2017).

P starvation directly inhibits both COX activity and the ATP synthase, resulting in low ATP/ADP ratios, while it promotes AOX activity, associated with higher NADH^+^H^+^/NAD^+^ ratios. This was demonstrated in *Phaseolus vulgaris* (Rychter and Mikulska, 1990; Rychter et al., 1992), *Catharanthus roseus* (Hoefnagels et al., 1993), *Chlamydomonas reinhardtii* (Weger and Dasgupta, 1993), *Lupinus albus* (Florez-Sarasa et al., 2014), and tobacco cell cultures (Parsons et al., 1999). The electron flow directed to the AOX pathway allows conserving the intercellular P pool (Theodorou et al., 1991; Parsons et al., 1999; Juszczuk et al., 2001; Juszczuk and Rychter, 2003; Day et al., 2004) but also allows NADH oxidation, produced during citrate synthesis, to maintain continuation of TCA cycle reactions (Vanlerberghe and McIntosh, 1996; Shane et al., 2004; Gupta et al., 2012; Florez-Sarasa et al., 2014). Moreover, under P limitation, AOX activity in roots seems positively correlated with synthesis and release of carboxylates (citrate and malate, López-Bucio et al., 2000; Veneklaas et al., 2003; Del-Saz et al., 2018).

The metabolic role of AOX remains unclear given the non-conserving energy of this pathway (Vanlerberghe, 2013). Other metabolic functions could be involved to sustain the basal metabolic process mainly based on a specific redox status (NAD(P)^+^/NAD(P)H^+^H^+^ cell pool). It has been also proposed that, concomitantly with AOX pathway, energy demand for plant metabolism is provided by fermentative activity (Mercy et al., 2017). AOX activity is promoted by accumulation of pyruvate, NADH^+^H^+^ and CO_2_ (Gonzàlez-Meler et al., 1996; Siedow and Umbach, 2000; Vanlerberghe, 2013), whose contents in roots are higher under low P (Juszczuk and Rychter, 2003). These three molecules can also favor fermentation activity (both lactic and alcoholic) while CO_2_ inhibites the COX pathway (Gonzàlez-Meler et al., 1996). Furthermore, malic enzyme converts malate to pyruvate, NADH^+^H^+^ and CO_2_, supplying fermentation pathway with suited substrates. It was shown that up-regulation of malic enzyme activity is associated with fermentation (Sakano, 2001), and is part of the alternative glycolytic pathway that is enhanced in P-deficient conditions (Schachtman et al., 1998; Plaxton and Carswell, 1999; Raghothama, 1999; Uhde-Stone et al., 2003a,b; Plaxton and Tran, 2011). Under P-deficient conditions, significant induction of fermentative related genes as alcohol dehydrogenase (Massonneau et al., 2001; Wu and Yang, 2003; Manan, 2012) and formate dehydrogenase (Herbik et al., 1996; Suzuki et al., 1998; Uhde-Stone et al., 2003b) has been shown, allowing the regeneration of NAD^+^ pool which avoids glycolysis inhibition (Tadege et al., 1999). Finally, increased ethanol concentration is observed with the application of COX inhibitors (Solomos and Laties, 1976; Kato-Noguchi, 2000), or antisense-induction of AOX genes in *Arabidopsis* under aerobic conditions (Potter et al., 2001). In mycorrhizal symbioses, the role of plant fermentation is not known but may contribute to fungal fitness as part of the favorable plant metabolism driven under low P.

#### Carbon Fluxes and Root Exudation Under Low P

Low P sensing *in planta* drives changes in carbon partitioning between shoots and roots. Sucrose is reallocated to the root where it participates to a rise in glucose concentration (Hammond and White, 2011; Lemoine et al., 2013), increasing availability of carbon sources for AM fungal uptake. Contents in many sugars, organic acids and several aminoacids are increased within roots and are released in the rhizosphere under P starvation, derived from blocked glycolysis and TCA cycle (Hoffland et al., 1992; Dakora and Phillips, 2002; Hernández et al., 2007; Yamakawa and Hakata, 2010; Carvalhais et al., 2011; Gupta et al., 2012). Such compounds can modulate AM spores germination and can mediate plant-AM fungi interactions at presymbiotic phases (Ratnayake et al., 1978; Graham et al., 1981; Hepper and Jakobsen, 1983; Gachomo et al., 2009). Many other compounds synthetized and released by plants under abiotic stress (notably low P) have known stimulatory impacts on mycorrhizal development: it is the case for H_2_O_2_ (Liu et al., 2012), for polyamines (El Ghachtouli et al., 1995; Wu et al., 2012), for certain flavonoids (Nair et al., 1997; Davies et al., 1999; Davies et al., 2005; Scervino et al., 2005) and other phenolic compounds (Fries et al., 1997) and probably most importantly strigolactones (Sun et al., 2014). Such molecules can also stimulate the release of diffusible factors from spore exudates, among which lipochitooligosaccharides and chitooligosaccharides (LCOs and COs), were characterized as so-called “Myc factors” (Nadal and Paszkowski, 2013; Schmitz and Harrison, 2014).

Plant-derived ET and diffusible factors present in germinating spore exudate (GSE) act antagonistically: compounds isolated from GSE (such as Myc factors) can stimulate mycorrhizal plant susceptibility, while ET inhibits GSE-induced symbiotic gene expression (Maillet et al., 2011; Mukherjee and Ané, 2011). Interestingly, pure LCO compounds extracted from *Bradyrhizobium japonicum* (similar to Myc factor found in GSE) applied to soybean leaves were shown to induce host stress response, activating AOX and repressing hormone-related components belong to GA signaling (Wang et al., 2012). Moreover, although composition of root exudates can vary depending on soil pH, plant species and plant age (Vierheilig et al., 2003; Badri and Vivanco, 2009; Tahat and Sijam, 2012; Balzergue et al., 2013), many of them released under low P (plant hormones as in particular SL, phenolic compounds, hydroxy fatty acids, glucosamine, specific aminoacids and sugars) were shown to act at pre-symbiotic stages (promoting spore germination, hyphal growth, hyphal branching), thus supporting the mycorrhizal symbiosis (Tamasloukht et al., 2003; Besserer et al., 2006, 2008; Nagahashi et al., 2010; Tawaraya et al., 2013; Nadal et al., 2017).

#### Hormone Interplay and Action Under Low P

Recognition and adaptation of plants to external metabolic stimuli is often mediated by phytohormone signaling. In particular, P starvation is associated with ABA accumulation (Mizrahi and Richmond, 1972; Vysotskaya et al., 2008), but also BRs (Nibau et al., 2008; Wang et al., 2014), IAA (Nacry et al., 2005), SL (Akiyama et al., 2005; Besserer et al., 2006, 2008; Foo et al., 2013b), and JA (Khan et al., 2016). As first well described hormonal regulation, plant mineral nutrition sensing is considered as the main driver modulating SL production (Umehara et al., 2010; Yoneyama et al., 2012) which is consistently promoted at P and nitrogen starvation (Bonneau et al., 2013; Foo et al., 2013b). For this reason, SL production at low P is considered as a plant strategy to recruit AM fungi for improving P uptake (Gu et al., 2011). P deficiency is also well correlated with low ET and reduced bioactive GA levels in roots, linked with an accumulation of DELLA proteins (Drew et al., 1989; Borch et al., 1999; Wu et al., 2003; Jiang et al., 2007; Kim et al., 2008; Devaiah et al., 2009; Hammond and White, 2011), although ET contributes to primary and adventitious root elongation (Nagarajan and Smith, 2012). The involvement of ABA signaling is linked with a positive cross-talk with JA and SL (López-Ráez et al., 2010; Lackman et al., 2011). JA and ABA can antagonize GA signaling *via* stabilization of DELLA proteins (Fu and Harberd, 2003; Yang et al., 2012) and can also negatively regulate SA signaling (Proietti et al., 2013; Manohar et al., 2017). Moreover, P starvation is known to decrease the synthesis of bioactive CKs, and some reports suggested that ABA participates to this inhibition (Pieterse and van Loon, 2004; Rouached et al., 2010; Nishiyama et al., 2011; Ha and Tran, 2014). It is well recognized that ABA regulates AOX gene expression and activity in plants (Finkelstein et al., 1998; Choi et al., 2000; Rook et al., 2006; Giraud et al., 2009; Lynch et al., 2012; Wind et al., 2012). In the work of Shen et al. (2003), abiotic stress and ABA were proposed to increase cytosolic levels of NADH^+^H^+^, which stimulate ROS production but also participate in the conversion of dihydroxyacetone phosphate to glycerol-3-phosphate, which then converts FAD to FADH_2_, providing electron flow toward the AOX pathway. Compelling evidence also demonstrates the role of ROS as a signal occurring in most abiotic and biotic stresses but also in symbiosis (Puppo et al., 2013; Ghosh and Xu, 2014) and in potentiating the ABA pathway (Kwak et al., 2003). Nutrient starvation (as low P) is usually associated with overproduction of H_2_O_2_ in roots (Shin and Schachtman, 2004; Shin et al., 2005; Cheeseman, 2007) able to upregulate AOX gene expression, protein content and activity (Juszczuk and Rychter, 2003; Yamaguchi-Shinozaki and Shinozaki, 2005; Ho et al., 2008; Wang et al., 2010). H_2_O_2_ originates mainly from NADPH oxidase and polyamine oxidase activities, both of which are induced by ABA (Wang, 2008; Liu et al., 2012). In particular, H_2_O_2_ is produced in arbuscules (Fester and Hause, 2005) and the use of scavengers (ascorbic acid or salicylhydroxamic acid) reduce both H_2_O_2_ and mycorrhizal development (Liu et al., 2012). Both ABA and ROS induce the production of JA (Howe, 2004; Yang et al., 2012) which plays an important role in plant defense.

### The Existential Problem of Mycorrhizal Fungi Under High Available P

The second condition that we describe is the situation of high available P, which is defined as concentrations known to inhibit mycorrhizal colonization in plants (Graham et al., 1981; Thomson et al., 1986; Balzergue et al., 2011) but also create the optimal state for plant growth in absence of AM fungi. Excluding the mycorrhizal context, several studies regarding plant physiology have been conducted under high (or optimal for the plant partner) P availability, and the metabolic frame is therefore relatively well known. In absence of stress, plant metabolism, especially photosynthesis, flows optimally, maximizing the energy yield (Nátr, 1992), associated with COX activity resulting in a high ATP/ADP ratio (Sluse and Jarmuszkiewicz, 1998; Day et al., 2004). This constant energy flux enables a steady state of metabolism (Kouchi and Yoneyama, 1984; Rontein et al., 2002). This steady state is an approximation that is always subjected to the so called “carbon economy” (Poorter et al., 1990) where the carbon accumulation, redistribution and utilization is continuously adjusted. However, a situation close to the steady state allows the plant to optimize many pathways involved in primary metabolism, providing important intermediates for other reactions (Krebs and Johnson, 1937). Part of the fixed carbon pass through the respiration while a fraction is then stored in lipid form in oil bodies (Napier et al., 1996).

Under high P, content and perception of some hormones is increased in roots such as ET, CK, GA, and SA (Drew et al., 1989; McArthur and Knowles, 1992; Jiang et al., 2007; Devaiah et al., 2009) and all can regulate positively photosynthetic pathways (Khan, 2004, 2006; Yaronskaya et al., 2006; Tholen et al., 2008; Rivas-San Vicente and Plasencia, 2011; Xie et al., 2016). GA controls sucrose synthesis by positively regulating fructose-1,6-bisphosphatase and sucrose phosphate synthase (Zamski and Schaffer, 1996) while SA promotes rubisco activity, chloroplastic structure (reviewed by Rivas-San Vicente and Plasencia, 2011) and net increase of CO_2_ assimilation (Fariduddin et al., 2003). GA and SA are known to have a reciprocal stimulation and it seems that both are involved in DELLA loss-of-function (Alonso-Ramírez et al., 2009a,b). A recent study indicated that GA signaling downregulates endogenous SL levels (Ito et al., 2017). High levels of GA, SA and ET repress synthesis and signaling of ABA and JA (Vlot et al., 2009). SL content in non-mycorrhizal roots is reduced under high P, and decreases also when P concentration increases locally (in arbuscocytes) after colonization is established (Foo et al., 2013b; Fusconi, 2014). GA biosynthesis and signaling is coupled with active COX pathway and both are key components to promote plant growth. *Arabidopsis* CYTc deficient mutant plant contains less bioactive GA, less ATP but elevated DELLA protein levels and similar observation was noticed when mitochondrial (COX pathway) inhibitors is applied in wild type (Racca et al., 2018). GA can upregulate cytochrome C gene expression involved in the COX pathway (Yang and Komatsu, 2004) and GA biosynthesis inhibitor mimicks the effect of CYTc deficiency (Racca et al., 2018). GA promotes respiration in *Amaranthus* while this effect is prevented by KCN (Loo et al., 1960). Robinson and Wellburn (1981) found that GA application increased the rate of NADH-dependant ATP formation, which is highly inhibited by cyanide (Cunningham, 1963) and can promote growth and lipogenesis (Yu et al., 2016). Then, SA was reported in many papers as promotor of AOX expression associated with increased protein levels, but it has actually no influence on its activity (Lennon et al., 1997; Simons et al., 1999). Instead, whole-cell tobacco respiration rate is enhanced when SA is applied at less than 1 mM (Norman et al., 2004). Finally, CKs may also act in mitochondria, by blocking the AOX pathway (Musgrave, 1994).

The negative influence of high P on mycorrhizal fungi is systematic and systemic as it was shown that foliar application of P can lead to the same depression phenomena (Breuillin et al., 2010). Knowledge of mechanisms involved in P inhibition on mycorrhiza remains fragmented, but deserves attention. Indeed, this element limits mycorrhizal inoculum performances under field conditions, where soils usually contain high amounts of available P (due to high application of P fertilization – Tóth et al., 2014). The statement that plants that are able to uptake P *via* the direct pathway do not need to establish mycorrhizal symbiosis appears simplistic, but underestimates the complex regulations that lead to AM inhibition. Actually, the hormonal composition (SA, GA and ET), enhanced under high P, creates an inhibitory context for mycorrhizal development, as detailed in section Phytohormones Influence the Mycorrhizal Symbiosis. From another point of view, the lack of mycorrhizal colonization was generally described as an economic determinism, so called “cost-benefit” trade between P uptake for plant and carbon delivery for AM fungi (Smith et al., 2011). From this perspective, it is recognized that plants usually adapt resource allocation to organ involved in mineral acquisition in order to stimulate their growth, where more energy is translocated from shoot to root under mineral shortage (such as sucrose, polyols such as mannitol and sorbitol and other oligosaccharides from raffinose family), but is linked with an impaired photosynthesis (Lemoine et al., 2013). By opposition, the source (shoot)-sink (root) balance is modified under high P: carbon sources become less available surrounding the mycorrhizal structures in roots, which may participate to reduce AM fungal growth and interface formation (arbuscules), thus repressing fungal P delivery to the plant. This goes also along with lower release of various molecules (sugars, amino acids, as well as some hormones such as SL) which can be recognized by mycorrhizal hyphae. Such lower release would limit root-fungus interactions. In addition to plant physiology, high P was also shown to inhibit directly spore germination and mycelium development *in vitro* (de Miranda and Harris, 1994; Olsson et al., 2002), limiting soil exploration and contact to the plant roots. However, it is not yet known, at the best of our knowledge, if AM fungi exudation of Myc factors is directly negatively affected under high P, like it is described for plant exudates. Importantly, AM fungi are partly aerobic organisms: this would mean that the processing of fungal energy from carbon sources must go concomitantly with higher O_2_ consumption to allow formation of ATP orientated by higher flow of electron toward the COX pathway. As a result, the inhibition of the COX pathway induced by application of KCN is associated with reduced mycorrhizal development under low P (Mercy et al., 2017). It can be questioned whether high P may induce a stronger source-sink (or competition) for oxygen, favoring plant cell energy which consequently becomes less accessible for the fungus.

P affects also the functionality of the symbiosis as mycorrhizal responses have been shown to be negatively correlated with increased P concentration (Smith and Smith, 2011a). This phenomena is not necessarily due to a decrease of carbon delivery to the fungus but more to a shift of P uptake between the direct and the mycorrhizal pathway. The current hypothesis states that AM fungi impair the direct pathway while the mycorrhizal P pathway does not compensate, probably due to a lower functional P transfer under high P (Smith and Smith, 2011b). To support part of this hypothesis, the down-regulation of the mycorrhiza-inducible P transporter genes PT4 have been described in several works for different plant species following application of high P concentration (Drissner et al., 2007; Breuillin et al., 2010). Moreover, it is possible that AM fungi try to prime a specific propitious plant metabolism (notably ISR based plant context, see section “Relationship Between Plant Defense Components and Mycorrhizal Symbiosis” above) but the full completion of this system permanently fails (leading therefore to persistent plant growth depression) due to the continuous and paradoxical responses from plant sensing and signaling under high available P.

## Conclusion and Perspectives

### Drawing Links Between AM Fungi, Plant Respiration, Phytohormones, Carbon Partitioning, and Plant Defense Upon Available P Concentration

#### Physiological Models of Plant Susceptibility to Mycorrhiza

**FIGURE 3:**
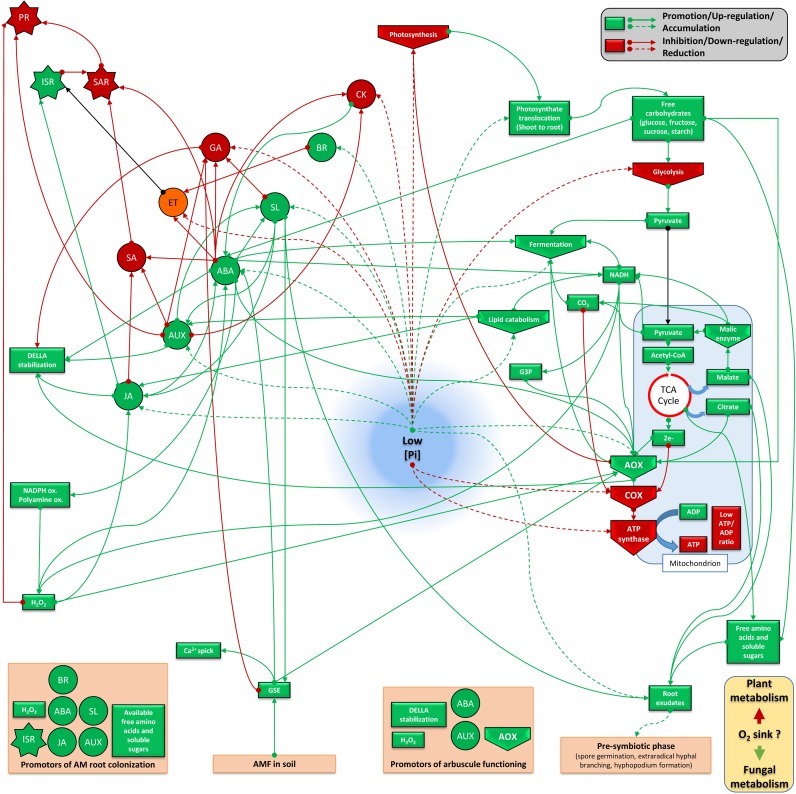
Plant metabolic orientation of hormone interplay, carbon partitioning and responses on mycorrhizal development under low available P. Box and arrow color indicate repression (red), or promotion (green). Orange boxes and black arrows are used for uncertain conditions. Based on the literature survey, mycorrhizal colonization is enhanced under low available P which goes together with the action of AM-promoting hormones (such as ABA, SL, and JA). This hormonal interplay is connected to a favorable metabolic frame which involves lower phytosynthetic activity, higher translocation of photosynthetats from shoots to roots, accumulation of sugars (reduced glycolysis flow and enhanced lipid oxidation), enhanced plant fermentation activity, cytosolic reductive potential (elevated NADH pool), electron partitioning which is orientated toward the alternative oxidase pathway, reduced ATP formation and ISR implementation. Root exudation of several sugars, aminoacids, some carboxylic acids and hormones (such as SL) participate to the molecular dialog with mycorrhizal fungi present in the rhizosphere. This can support physical contact with the root by stimulating hyphal branching and to induce plant responses by promoting Myc factor release from germative spore exudates. It is questioned if this metabolic flux is accompanied by lower oxygen consumption by plant cells, which may become more available for the fungus (as aerobic organism) under low P. ABA, abscisic acid; JA, jasmonate; GA, gibberellins; SA, salicylic acid; SL, strigolactones; ET, ethylene; CK, cytokinins; IAA, auxins; BR, brassinosteroids; PR, pathogenesis related protein; ISR, induced systemic response; SAR, systemic acquired resistance; AOX, alternative oxidase; COX, cytochrome oxidase; CytC, cytochrome C; TCA, Krebs cycle; NADPH ox., NADPH oxydase; polyamine ox., polyamine oxydase; GSE, germinative spore exudate; G3P, Glycerol 3-phosphate.

**FIGURE 4:**
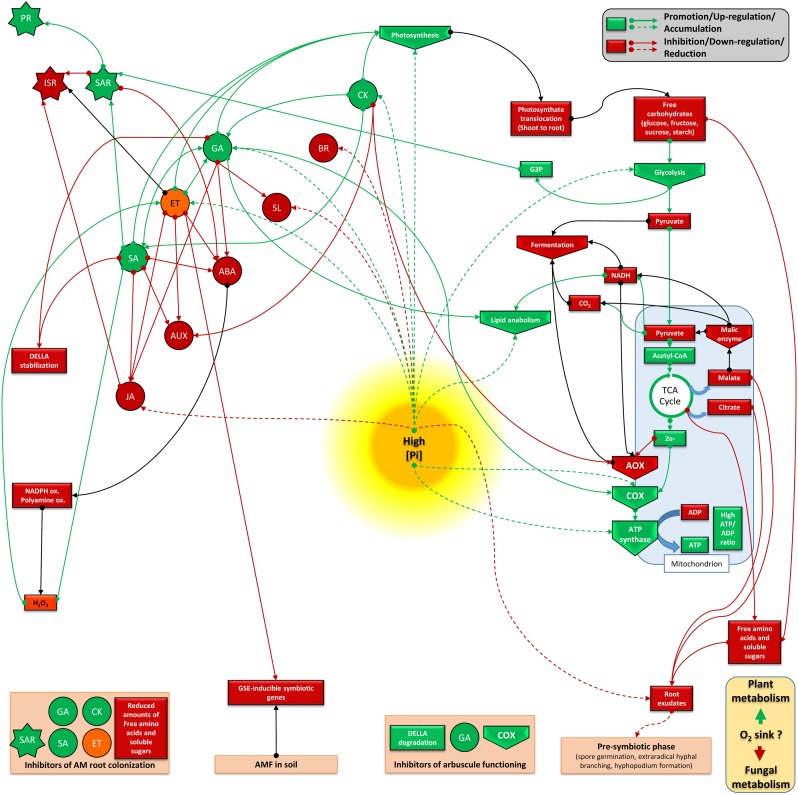
Plant metabolic orientation of hormone interplay, carbon partitioning and responses on mycorrhizal development under high available P. Box and arrow color indicate repression (red), or promotion (green). Orange boxes and black arrows are used for uncertain conditions. Based on the literature survey, high available P affects mycorrhizal performances mainly due to the activity of mycorrhiza-inhibiting hormones (such as GA, SA, and ET), and in which SAR is potentialized. This goes together with a non-favorable metabolic frame which seems connected to an enhanced photosynthesis activity, lower translocation of photosynthetats from shoots to roots, a continuous flow of sugars processed via glycolysis, lipogenesis, and TCA, but also reduced fermentation activity and higher oxidative potential (reduced NADH^+^H^+^ cytosolic pool). In this system, lower free amounts of compounds (sugars, aminoacids, SL, and carboxylate acids) are released in the root exudate, thus reducing possible molecular dialog between AM fungi and plant root. In addition, high P favors electron partitioning toward the plant COX pathway, thus participating to ATP formation. It is questioned if this metabolic flux is accompanied by higher oxygen consumption by plant cells, which may become less available for the fungus (as aerobic organism) under high P. ABA, abscisic acid; JA, jasmonate; GA, gibberellins; SA, salicylic acid; SL, strigolactones; ET, ethylene; CK, cytokinins; IAA, auxins; BR, brassinosteroids; PR, pathogenesis related protein; ISR, induced systemic response; SAR, systemic acquired resistance; AOX, alternative oxidase; COX, cytochrome oxidase; CytC, cytochrome C; TCA, Krebs cycle; NADPH ox., NADPH oxydase; polyamine ox., polyamine oxydase; GSE, germinative spore exudate; G3P, Glycerol 3-phosphate.

Studying phytohormone interrelationships is always delicate, since there exist fine and complex regulations which depend on tissues and the plant physiological stage. Nevertheless, we propose two models (Figures 3, 4) that sum up two antagonistic metabolic situations according to P levels. This allows to distinguish two groups of acting hormones: (i) a first group (JA, ABA, IAA, SL, and BR) is involved in signaling under low P (Figure 3), has known overall promoting activities toward AM fungi development and seems to be linked potentially to the ISR system. The metabolic state comprises higher fermentation activities, increased free cytosolic amounts and root release of sugars, aminoacids and carboxylate acids, promotion of lipid catabolism, higher cytosolic reductive potential and the involvement of AOX pathway; (ii) a second group of hormones (GA, SA, ET, and potentially CK) is active under high P (Figure 4), has inhibitory impacts on AM fungi development and seems to be linked to the SAR system. It involves repression of fermentation, implementation of lipid anabolism, a higher cytosolic oxidative potential and the involvement of the COX pathway.

Consequently, the processing (uptake and metabolic assimilation) by AM fungi of the energy sources provided by the plant (sugars, lipids and maybe other compounds as products of fermentation) may depend on the presence of a surrounding metabolic context that integrates favorable signaling (partly mediated by hormones and reductive potential, upon likely available oxygen fluxes). Our literature survey suggests that plant AOX pathway may play one central role in the implementation of a specific metabolism, which occurs consequently to environmental stimuli (stress). Obviously, phytohormone regulations and electron flow partitioning between AOX and COX is dynamic in time and space and this must be taken into account during mycorrhizal development under normal conditions. The AOX pathway coupled with fermentative processes is probably a main component during the first stages of mycorrhizal development, explaining perhaps the well known stunt phenomenon which follows plant inoculation. Successively, electron route to AOX pathway and the related plant metabolism is very likely shifted toward the COX pathway (Del-Saz et al., 2017), as a consequence of increasing level of P delivered by the mycorrhizal pathway. Therefore, the metabolic context occurring at later stages of the mycorrhizal symbiosis may shift partly toward the model described in the Figure 4. This would fit with the known increased photosynthetic activity, reduced root exudation of compounds and electron partitioning through the COX pathway, as responses that AM fungi trigger in plant. This shift might also participate in the autoregulation of mycorrhization, a mechanism which prevents subsequent mycorrhizal development following a first plant inoculation with AM fungi (Vierheilig et al., 2000). ABA and JA seem not to take part in this phenomenon, while AUX and CK might be involved (Meixner et al., 2005; Wang et al., 2018), but the role of other AM-inhibiting hormones remain to be studied.

#### Gaps and Limits of Hormonal Studies in Mycorrhiza

We attempted to link pieces of the puzzle that shows fragments of the final picture but does not allow yet full understanding of the whole composition. Many efforts are still needed to deeply investigate the role of phytohormones with respect to AM fungi and plant metabolic adaptation. To date, the use of different plant species, their related mutants, and the different application ways of phytohormones create sometime discrepancies in the final outcomes. It is not trivial to interprete phenotypic and molecular data only related to the specific mutation which requires several internal controls. When mutant plants are able to survive, general metabolic and/or signaling pathways are likely differentially adapted surrounding the deleted or overexpressed targeted gene(s), thus participating at a whole on the responses on the mycorrhizal behavior. It appears also that some methods might create biases. For example, soil hormone application can already affect the mycorrhizal propagules during pre-symbiotic phase, and the eventual release of hormones by AM fungi could interfer with phenotypic responses of mutant plants. Production of several hormones (as SL, ET, JA, SA, and BR) by AM fungi remains unknown, while they may potentially participate in fungal spread within roots like pathogenic fungi (Chanclud and Morel, 2016). For example (but not exclusively), although SL biosynthesis seemed never investigated in any other organism than plants, it would be interesting to check the eventual ability of AM fungi to secrete this hormone. This might explain why arbuscules are still well formed within different SL deficient mutant plants (Kobae et al., 2018). In addition, data are sparse regarding influence of hormones on mycorrhizal behavior (pre-symbiotic and symbiotic stages) under *in vitro* conditions, and reports seem lacking for some of them (such as JA, SA, or BR). Moreover, most of the studies conducted with phytohormones within endomycorrhizal systems were set under low P, as it represents suitable conditions to investigate fungal phenotypes. Nonetheless, we recommand to perform more assays with different P concentrations (but also with other elements), in order to better define the mode of action of each hormone. Then, it could be interesting to report mycorrhizal phenotype traits under axenic and monoxenic systems that combine a given hormone in presence of various carbon sources. Despite several sugar transporters were localized in arbuscules but also in hyphae of the extraradical mycelium (Helber et al., 2011), assimilation and processing of carbon sources could require specific signals for generating efficiently fungal energetic fluxes, thus supporting growth and sporulation. In the future, the creation of a shared study platform would be useful, where application of all phytohormones are standardized under common conditions, taking care to evaluate not only the mycorrhizal phenotypic traits, but also metabolic plant adaptation.

Some aspects were not addressed in this review. This concerns the sporulation process, because the metabolic context that drives this specific fungal developmental step belongs to physiological changes that take place following root colonization at late stage. Moreover, the relationship between hormone interplay and mycorrhizal spore formation is only poorly investigated, despite this structure could reflect the energy status of the fungus (Supplementary Material). Information about the importance of fatty acid transfer as source of energy from plant to AM fungi did not find much space in our scheme, despite recent findings about its potential role (Keymer et al., 2017; Luginbuehl et al., 2017; Roth and Paszkowski, 2017). This field of research can represent a breakthrough in the understanding of AM symbiosis, but to date, almost no information is available regarding AM fungal phenotypes related to regulations between plant lipid energetic metabolism, hormone interplays and P levels, at the best of our knowledge. Plant *RAM1*, *RAM2*, *FatM*, and *STR/STR2* were proposed to act as operational unit to synthetize and deliver fatty acid to AM fungi (Keymer et al., 2017). In particular, reduced mycorrhizal development was demonstrated in *ram1/2* deficient mutant plants (Gobbato et al., 2012; Keymer et al., 2017). To illustrate first links toward lipid pathway in relation with fungal energetic needs, it was shown that application of Myc factors can upregulate *RAM1/2* expressions (Jiang et al., 2018), while GA (inhibiting mycorrhizal development) can downregulate *RAM2* expression (Takeda et al., 2015). However, a question raises whether the reduced mycorrhizal colonization in *ram2* mutant plants is due to the specific mutation, or due to a side effect from mycorrhizal inhibiting hormones (such as GA), that might be overexpressed as part of a signaling adaptation.

We are aware that hormonal interplays and regulations at different environmental conditions and in various plant species are much more complex than our models suggest, which remain largely incomplete. Therefore, next steps should attempt to validate or reject some of the hypotheses deduced from the models with further investigations. The effort that we made was an attempt to define a consensus, but also to propose several research topics aiming to elucidate some fundamental aspects of the endosymbiotic relationship which are still not fully understood and exploited.

### Perspectives for Application: The Induction Methods for Mycorrhiza

**FIGURE 5:**
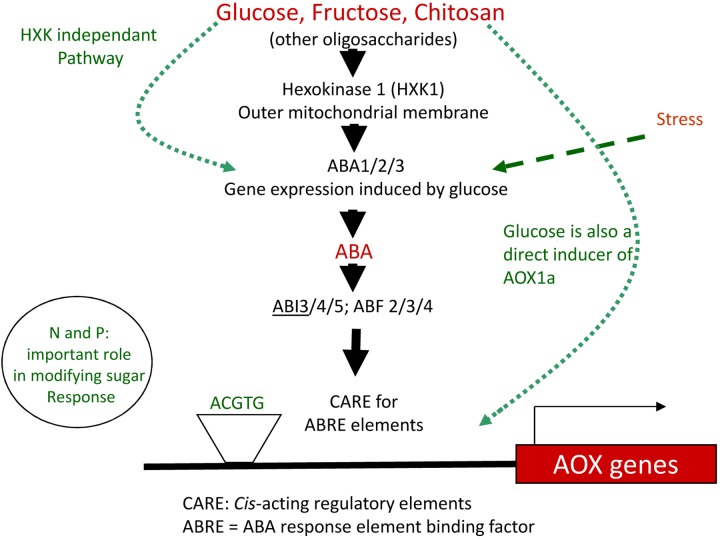
Theoretical scheme of the oligosaccharides signaling on alternative oxydase pathway, via ABA-dependant and independent regulation.

According to our model, AM fungi development *in planta* seems to be promoted by the occurrence of ISR and its related signaling, prior to AM fungi contact. The induction of ISR or SAR system can be primed by application of specific elicitors for one or the other system. The use of specific molecules able to generate a favorable metabolic context to promote an effective colonization can therefore be proposed to master mycorrhizal inoculum applications under practical field condition or other research area as degraded land restauration. In this view and among those stimulatory molecules, potential affordable strategies exist from the application at low doses (seen as signal) of oligosaccharides on plants. Interestingly, oligosaccharides were shown since some decades to act as elicitors and therefore implement specific plant defense responses against biotic but also abiotic stress (Trouvelot et al., 2014). Oligosaccharides possess several advantages, such as being cheap and available, non-toxic, biodegradable, easy to use and not classified as phytohormones (whose field application is highly restricted in Europe). Linking plant respiration and plant priming, the idea consists to induce a specific transient plant stress, by targeting the AOX pathway and its related metabolism, as it was shown to play a crucial role in arbuscule formation and positive mycorrhizal response (Mercy et al., 2017). Sugar signaling can promote AOX pathway directly (Li et al., 2006) or indirectly via the ABA signaling. In this last case, sugar recognition by the hexokinase 1 (Ramon et al., 2008), present on the outer mitochondrial membrane, initiates ABA synthesis (Cheng et al., 2002) and then stimulates the AOX gene expression *via* transcription factors (Finkelstein et al., 1998; Rook et al., 2006; Ho et al., 2008; Giraud et al., 2009; Millar et al., 2011). Although this signaling scheme (illustrated Figure 5) remains hypothetical, first trials using application (soil or on leaves) of low dose of oligosaccharides (such as glucose, fructose, and xylose) show possibilities to improve mycorrhizal development and responses under various P concentrations and in several plant and AM fungal species (Lucic and Mercy, 2014 – Patent application EP2982241A1; Bedini et al., 2017; Lucic-Mercy et al., 2017). Since the same compounds were termed initially as elicitors, related to the implementation of plant defense upon pathosystems but can also promote mycorrhizal performances, we propose to use rather the appellation “inducer” (or Mycorrhizal Helper Signaling Molecules), which defines signaling molecules that are intended to act specifically as stimulants in endomycorrhizal systems. In the same way, it would be also interesting to check if the mycorrhizal susceptibility is connected with plant species and cultivars that harbor naturally preferential mitochondrial electron partitioning toward the AOX pathway (as one metabolic selection trait). Such an approach may allow then to define interesting strategies for breeders, in order to orientate the plant selection in view to optimize mycorrhizal interactions in crops.

## Author Contributions

LM took the lead in writing the manuscript and conceived the main subject of this review. AB and LM drafted the manuscript. CS, PF, and EL-M supervised the writing, critically revised the manuscript, and contributed to its final version. All authors approved the final version of the manuscript.

## Conflict of Interest Statement

The authors declare that the research was conducted in the absence of any commercial or financial relationships that could be construed as a potential conflict of interest.

## Footnotes

1 http://glomeromycota.wixsite.com/lbmicorrizas/copia-informacoes-gerais-lista

## Supplementary Material

TABLE S1 List of references and overview of hormone on AM symbiosis.
